# Identification of Potential Inhibitors from Pyriproxyfen with Insecticidal Activity by Virtual Screening

**DOI:** 10.3390/ph12010020

**Published:** 2019-01-25

**Authors:** Ryan da Silva Ramos, Josivan da Silva Costa, Rai Campos Silva, Glauber Vilhena da Costa, Alex Bruno Lobato Rodrigues, Érica de Menezes Rabelo, Raimundo Nonato Picanço Souto, Carlton Anthony Taft, Carlos Henrique Tomich de Paula da Silva, Joaquín Maria Campos Rosa, Cleydson Breno Rodrigues dos Santos, Williams Jorge da Cruz Macêdo

**Affiliations:** 1 Postgraduate Program in Biotechnology and Biodiversity-Network BIONORTE, Federal University of Amapá, Macapá, 68903-419 Amapá, Brazil; ryanquimico@hotmail.com (R.d.S.R.); alexrodrigues.quim@gmail.com (A.B.L.R.); ericamrabelo@gmail.com (E.d.M.R.); williamsmacedo@yahoo.com.br (W.J.d.C.M.); 2 Laboratory of Modeling and Computational Chemistry, Department of Biological and Health Sciences, Federal University of Amapá, 68902-280 Macapá, AP, Brazil; josivan.chemistry@gmail.com (J.d.S.C.); camposchemistry@gmail.com (R.C.S.); vilhenac@hotmail.com (G.V.d.C.); 3 Laboratory of Molecular Modeling and Simulation System, Federal Rural University of Amazônia, Capanema, 68700-030 Pará, Brazil; tomich@fcfrp.usp.br; 4 Computational Laboratory of Pharmaceutical Chemistry, Faculty of Pharmaceutical Sciences of Ribeirão Preto, 14040-903 São Paulo, Brazil; 5 Laboratory of Arthropoda, Federal University of Amapá, 68902-280 Macapá, AP, Brazil; nonatoiepa@hotmail.com; 6 Brazilian Center for Physical Research, Urca, 22290-180 Rio de Janeiro, Brazil; catff@terra.com.br; 7 Department of Pharmaceutical Organic Chemistry, University of Granada, Granada 18071, Spain; jmcampos@ugr.es

**Keywords:** acetylcholinesterase, juvenile hormone, PASS, molecular docking

## Abstract

*Aedes aegypti* is the main vector of dengue fever transmission, yellow fever, Zika, and chikungunya in tropical and subtropical regions and it is considered to cause health risks to millions of people in the world. In this study, we search to obtain new molecules with insecticidal potential against *Ae. aegypti* via virtual screening. Pyriproxyfen was chosen as a template compound to search molecules in the database Zinc_Natural_Stock (ZNSt) with structural similarity using ROCS (rapid overlay of chemical structures) and EON (electrostatic similarity) software, and in the final search, the top 100 were selected. Subsequently, in silico pharmacokinetic and toxicological properties were determined resulting in a total of 14 molecules, and these were submitted to the PASS online server for the prediction of biological insecticide and acetylcholinesterase activities, and only two selected molecules followed for the molecular docking study to evaluate the binding free energy and interaction mode. After these procedures were performed, toxicity risk assessment such as LD_50_ values in mg/kg and toxicity class using the PROTOX online server, were undertaken. Molecule ZINC00001624 presented potential for inhibition for the acetylcholinesterase enzyme (insect and human) with a binding affinity value of −10.5 and −10.3 kcal/mol, respectively. The interaction with the juvenile hormone was −11.4 kcal/mol for the molecule ZINC00001021. Molecules ZINC00001021 and ZINC00001624 had excellent predictions in all the steps of the study and may be indicated as the most promising molecules resulting from the virtual screening of new insecticidal agents.

## 1. Introduction

*Aedes aegypti* is the main vector of yellow fever, dengue, chikungunya, and Zika virus in all tropical and subtropical areas of the planet [1,2]. According to the World Health Organization [3], dengue is a viral disease with greater spread transmitted by mosquitoes over the last 50 years and infects around 50 to 100 million people annually, exposing the risk of death to almost half of the world population in regions endemic of the virus [4].

According to Brazilian Ministry of Health data [5], 589,107 cases of classical dengue fever and 1297 cases of dengue hemorrhagic fever were reported in the country until 2013, of which 235 were fatal. The control strategies of the main dengue vector are based on the use of chemical and biological products, integrated with environmental management software [6]. It is a growing health problem that is estimated to pose a risk to 2.5 billion people, mainly affecting countries in South and Southeast Asia [7].

The crystal structure of the *Drosophila melanogaster* acetylcholinesterase enzyme (AChE) was determined by Kroupova et al. (2018) [8], but currently, there does not exist any AChE structure for the mosquito *Aedes aegypti* available in the Protein Data Bank (PDB). *D. melanogaster* AChE has 37–39% amino acid sequence identity to the corresponding enzymes of *Anopheles gambiae* and *Ae. aegypti*, respectively; notably, the mosquito (*Ae. aegypti)* and human acetylcholinesterase enzymes exhibit slightly increased sequence identity 48–49% [9,10,11]. This justifies several in vitro studies of acetylcholinesterase activity in order to confirm such enzymatic inhibition; as an example, Botas et al. (2017) [10] carried out a study on the chemical composition, anticholinesterase activity, and nanoemulsions of limonene as a larvicidal agent for the control of *Ae. aegypti* (Diptera: Culicidae) of the essential oil of *Baccharis reticularia* DC. In this study, the essential oil was able to inhibit the enzyme acetylcholinesterase with an IC_50_ value of 301.9 μg/mL, demonstrating moderate anticholinesterase activity when compared to other oils of Asteraceae species.

Essential oils are mixtures of volatile compounds that can be produced by plants as part of their chemical defense against phytophagous invertebrates, mainly by enzyme inhibition [11]. Despite the fact that several volatile terpenoids (mono and sesquiterpenes) present insecticidal activity by acetylcholinesterase enzyme inhibition, some of them may have activity modulated by the presence of other substances, including those of complex mixtures [12,13,14]. Hence the great need to carry out new research with isolated or synthesized compounds to understand the elucidation of insecticide mechanism.

Among the several mechanisms of action, the inhibition of the insect-acetylcholinesterase enzyme stands out as a promising method of insecticide control. Inhibitors of this type of mechanism affect the transmission of nerve impulses by accumulating acetylcholine in the neuromuscular tissue of insects, causing paralysis and then death [15]. Therefore, the AChE inhibitors’ discovery is an important task, in which the development of insecticides based on natural molecules play a fundamental role [16,17]. Therefore, it can be affirmed that there is a relation that the chemical insecticides described in the literature act in the central nervous system in different target sites, for example, acetylcholinesterase (AChE) and juvenile hormone (JH) [15,16,17].

The JH analogues represent a class of insecticides that have been specifically designed to disrupt unique regulated endocrine processes and are a key regulator of insect development and breeding [14]. In adult mosquitoes, it is essential for ovary maturation and normal male reproductive behavior, but as the distribution and activity of JH are regulated is unclear after secretion [15].

**Figure 1:**
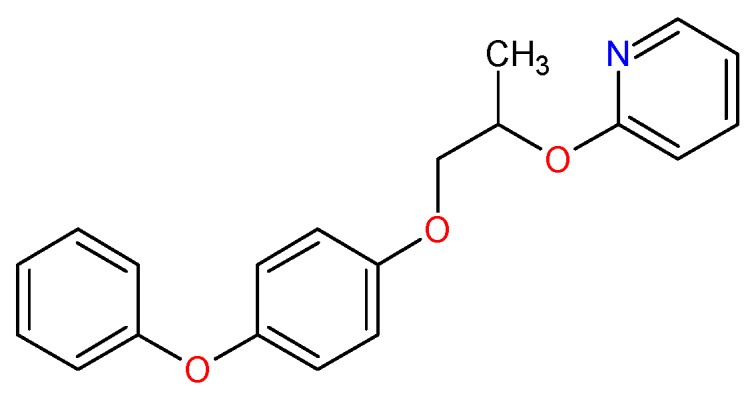
2D structural formula of pyriproxyfen (2-[1-methyl-2-(4-phenoxyphenoxy) ethoxy] pyridine).

The insecticide pyriproxyfen (2-[1-methyl-2-(4-phenoxyphenoxy) ethoxy] pyridine) [9], as shown in Figure 1, is an aromatic compound, non-terpenoidal hormone, a potent suppressor of embryogenesis of insects at the adult stage, and belongs to the chemical group pyridyloxypropyl ether, synthesized and developed by Sumitomo Chemical Co., Ltd. in the 1990s [18,19,20].

Pyriproxyfen is a phenyl carbonyl derivative in which a part of the aliphatic chain has been replaced by pyridyl oxyethylene. It is a copycat of a powerful juvenile hormone that affects the hormonal balance in insects, resulting in strong suppression of embryogenesis, adult metamorphosis, and inhibiting the development of adult insect characteristics (egg, wings, maturation of the reproductive organs, and external genitalia), keeping it “immature” (larva or pupa). It is often used as a biorational pesticide, i.e., a naturally occurring pesticide that ostensibly has limited or no effect on the environment or beneficial organisms. The biorational pesticides, better known as biopesticides, are derived from biological sources; for example, viruses, bacteria, fungi, etc., or from biochemicals such as insect growth regulators (IGRs) [21,22,23]. 

In this study, we sought to obtain new molecules with potential insecticidal activity against *Ae. aegypti* via virtual screening. Pyriproxyfen was chosen as a template compound to search molecules in the database Zinc_Natural_Stock (ZNSt) with structural similarity using ROCS (rapid overlay of chemical structures) and EON (electrostatic similarity) software. Subsequently, in silico pharmacokinetic and toxicological properties were used, as well as the online PASS server for the prediction of biological insecticide and acetylcholinesterase activities, followed by the molecular docking study to evaluate the binding free energy and interaction mode of the screened compounds.

## 2. Results and Discussion

### 2.1. Pharmacokinetic and Toxicological Properties

The pharmacokinetic properties are described in Table 1 for 14 molecules selected from virtual screening using the Rapid Overlay of Chemical Structures method implemented in the ROCS software followed by electrostatic similarity calculation using the EON software. The “star” parameter means the number of property values or descriptors that are outside the 95% range of similar values for known drugs. A large number of “stars” suggest that a molecule is less drug-like than molecules containing few stars.

After thorough evaluation of Absorption, Distribution, Metabolism, and Excretion (ADME) parameters, such as MW (molecular weight), QP logKp (predictable permeability of the skin), HB donor (estimated number of hydrogen bonds that would be donated by the solute to water molecules in aqueous solution), HB acceptor (estimated number of hydrogen bonds that would be accepted by solute molecules of water in aqueous solution); according to rule of five, all the compounds satisfied the conditions [22,24]. All molecules tested in the present study exhibit hydrogen bonding and also display hydrophobic interactions with corresponding amino acids, according to the molecular docking simulations performed here (see Section 2.3). All the molecules investigated here did not present any violation, except for the template compound (pyriproxyfen), but they are in accordance with Lipinski’s rule of five, and only five molecules violated Jorgensen’s rule of three, which is in accordance with studies carried out by Gaddaguti et al. (2016) [25]. Compounds with less, or preferably no violations, of these rules are more likely to be administered/available by the oral route [25]. 

The toxicological properties of the molecules with toxicity alarms are shown in Table 2. It was noted that the pyriproxyfen in the toxicity analysis did not indicate any alert, a fact that can be justified considering the low concentration in which it acts in the active site [25,26,27].

Analysis of the toxicological properties allowed the observation that of the 14 molecules, nine presented toxicity alerts characterized as plausible or acceptable and five (ZINC13537284, ZINC00001021, ZINC01530718, ZINC00000257, and ZINC00001624) did not present any type of alert.

Hepatotoxicity of steroid hormones describes the pathological conditions associated with the administration of these compounds and includes intrahepatic cholestasis, vascular disorders, and neoplasms [27]. The mechanism of cholestasis induced by steroids is believed to include a measure of intrinsic toxicity. The structural similarity with endogenous bile acids led to the suggestion that competition with bile acid transport could contribute to the observed effect [28,29].

The skin sensitizing activity results from the phenyl acylation of skin protecting esters, following the nucleophilic attack of skin proteins on the carbonyl of the ester group [30,31]. Activity for such compounds has been demonstrated in various skin sensitization assays, including the guinea pig maximization test [32] and adjuvant single injection tests. Skin sensitization in humans has also been described [33].

Related evidence has been extensively reviewed for the binding of 17-β-estradiol to the estrogen receptor [34]. The receptor seems to involve the ligand and, as a result, all four rings of the steroid nucleus contribute significantly to the binding. In addition, the hydrogen bond between the receptor and the phenolic and β-hydroxyl groups also plays a role. The binding is generally reduced by the introduction of polar substituents into the structure, while hydrophobic groups are tolerated at various positions subject to steric constraints. In vitro receptor binding assays suggest that the phenolic hydroxyl group is more important than the 17-β-hydroxyl group in terms of estrogen receptor binding affinity [35].

The alert describes the teratogenicity of 17-β-estradiol and its analogs. These compounds are potential ligands for the estrogen receptor (ER) and may cause birth defects as a result of their interaction with that receptor. In the present study, 17beta-estradiol [36,37], ethinyl estradiol [38], and dipropionate of estradiol [36,37] produced malformation in the reproductive organs of both male and female offspring when administered orally or subcutaneously to the mother during the second half of gestation (including the period of sexual differentiation).

The alert describes the genotoxicity of alkylating agents wherein the carbon containing the functional group is a primary or secondary alkyl carbon atom. In addition to the alkyl halides, it also includes the alkyl, sulphonated, and sulfonated sulfonates which lack a hydroxyl group directly attached to the sulfur [39]. Alkyl halides are electrophilic species capable of directly alkylating the DNA. Therefore, many compounds are mutagenic in the Ames test in the presence and absence of the S9 mixture, particularly *Salmonella typhimurium* in strains TA100 and TA1535 [40,41,42].

The in vitro prediction of inhibition of hERG channels is one of the toxicological factors that are related to side effects that new drug candidates may present. The hERG channels belong to the Shaker family, a subtype of gene that belongs to a subunit of potassium channels that are voltage controlled. Its inhibition or alteration causes prolongation of the ventricular repolarization phase of the heart and may still cause cardiac arrhythmias [43,44].

### 2.2. Biological Activity Prediction

**Figure 2:**
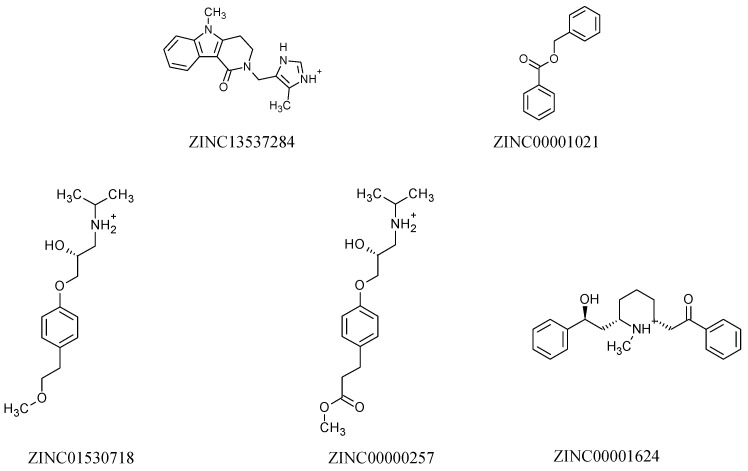
2D structures of selected molecules with good pharmacokinetic and toxicological profiles.

Using virtual screening with the ROCS and EON software, respectively, we have selected the top-ranked hits, which were subsequently subjected to pharmacokinetic predictions, resulting in fourteen molecules with a good pharmacokinetic profile, and only five showed no toxicity alarm. The selected structures with good pharmacokinetic and toxicological profiles are visualized in Figure 2. Prediction of the biological activity using the PASS web server [45] resulted in the data shown in Table 3. The reference compounds (pyriproxyfen, I40, GNT, and JHIII) showed insecticidal activity, corroborating the results of the literature [17,18,19,20,21,22,23]. 

Molecules ZINC00001021 and ZINC00001624 showed predictions satisfactory for insecticidal activity, acetylcholine antagonist and acetyl esterase inhibitor, all with Pa > 0.4, being similar to other known bioactive compounds, when Pa > Pi, as shown in Table 3.

### 2.3. Molecular Docking Study

In order to validate the molecular docking method used here, the compounds with the crystallographic information were subjected to the development of docking until the spatial conformation was found using AutoDock 4.2/Vina 1.1.2 software, via graphical interface PyRx by comparison with the original crystallographic structure of the acetylcholinesterase (AChE) inhibitors (PDB IDs 1QON and 4EY6) and the juvenile hormone III structure (PDB ID 5V13).

**Figure 3:**
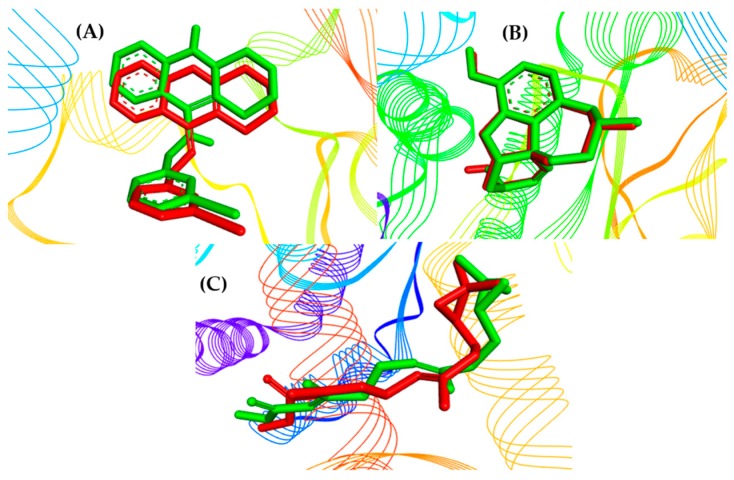
Superpositions of crystallographic ligands poses (in green) with the calculated poses (in red): (**A**) I40, (**B**) GNT, and (**C**) JHIII.

Retrieving the pose of each AChE inhibitor (I40, GNT, and JHIII), it was possible to perform validation of the molecular docking protocols used here, calculating root-mean-square deviations (RMSD) of 0.82, 0.37, and 1.27 Å, respectively. According to Gowtham et al. (2008) [46] and Hevener et al. (2009) [47], the binding mode predicted using docking indicates that when the RMSD is less than 2.0 Å regarding the crystallographic pose of a respective ligand, validation is considered satisfactory. The best results can be seen in Figure 3.

The molecular docking method used here identified a conformation that allows the ligand to also bind the residues of the I40-active sites (PDB ID 1QON) around the α-helix between amino acid residues Tyr370–Tyr374 and around the β-sheet between amino acid residues Ile82–Thr85 and Val478–His480. For the ligand, it is possible to see hydrogen bonds in common with residues Tyr370 and His480. There was also a hydrophobic interaction with residues Tyr71, Trp83, Tyr370, Phe371, and Leu479, corroborating the studies of Harel et al. (2000) [48].

Residues of the GNT-active sites (PDB 4EY6) were located around the α-helix between amino acid residues 336–338 and on β-sheet between the amino acid residues 85–87, 121–124, and 202–203. For the ligand it was possible to see hydrogen bonds in common with the residues Tyr124, Glu202, and Ser203. There were also hydrophobic interactions with residues Trp86, Gly121, and Tyr337, as identified in studies conducted by Gaddaguti et al. (2016) [25].

Interactions with the JHIII site (PDB 5V13) were located around the α-helix between the amino acid residues Ser30-Ala38, Arg45-Glu51, Val60-Gln71, Phe123-Leu130, Val132-Arg136, Leu138-Arg143, and Val280-Trp286 for the β-sheet between the amino acid residues Pro52-Pro55, Tyr72-Val73, Thr144-Val145, and Arg276-Gln279. For the ligand, it was possible to observe hydrophobic type interactions with all amino acid residues, according to studies carried out by Olmstead et al. (2003) [20].

**Figure 4:**
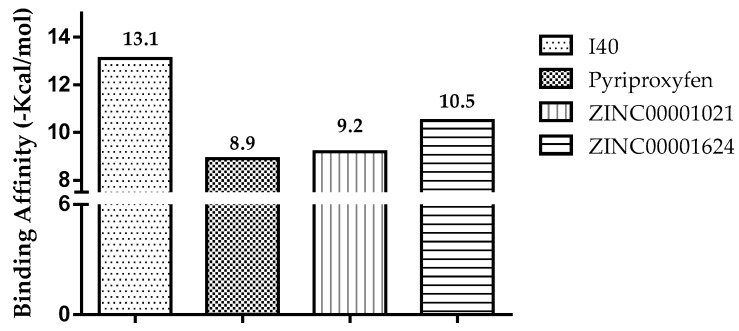
Results of binding affinity of the compounds with insect acetylcholinesterase (*Drosophila melanogaster* organism), Protein Data Bank (PDB) ID 1QON.

In order to evaluate if the interactions had a higher binding affinity than the specific ligand (I40, GNT, and JHIII) for acetylcholinesterase from different organisms (*Drosophila melanogaster* and *Homo sapiens* organism) and mosquito juvenile hormone (*Aedes aegypti* organism), it was observed that of the five compounds submitted to docking, only two presented values higher than or equal to the negative controls used here. Compound ZINC00001624 has a binding affinity of −10.5 kcal/mol, followed by ZINC00001021 with −9.2 kcal/mol compared to the controls I40 and pyriproxyfen (PDB ID = 1QON, *Drosophila melanogaster* organism), according to Figure 4.

The I40 exhibited a binding affinity of −13.1 kcal/mol higher than pyriproxyfen of −8.9 kcal/mol. However, the compound ZINC0001624 showed a binding affinity value of −10.5 kcal/mol higher than the controls used in molecular docking. Thus, by comparing the compound ZINC0001624 to the I40 control, a difference of ±2.6 kcal/mol was observed, whereas a variation of ±1.6 to ±1.3 kcal/mol was observed for the others, as shown in Figure 4.

**Figure 5:**
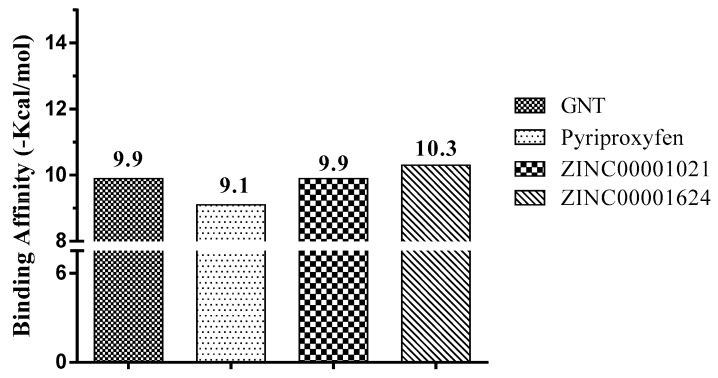
Results of binding affinity of the compounds with human acetylcholinesterase (*h*AChE), PDB ID 4EY6.

In the human *h*AChE, the inhibitors showed higher binding affinity and free energy values compared to the pyriproxyfen used in the molecular docking procedure performed here. These values corroborate the obtained similarity in the amino acid residue sequence in which the compound ZINC00001624 showed a high affinity value of −10.3 kcal/mol, followed by ZINC00001021 with −9.9 kcal/mol, according to Figure 5.

GNT exhibited a binding affinity of −9.9 kcal/mol and pyriproxyfen, of −9.1 kcal/mol. However, compound ZINC0001624 has a binding affinity value of −10.3 kcal/mol, hence higher than the obtained for the controls docked here. Thus, by comparing the compound ZINC0001624 to the GNT control, a difference of ±0.4 kcal/mol was observed, whereas for the others, a variation of ±0.6 to ±0.4 kcal/mol was observed, as shown in Figure 5.

**Figure 6:**
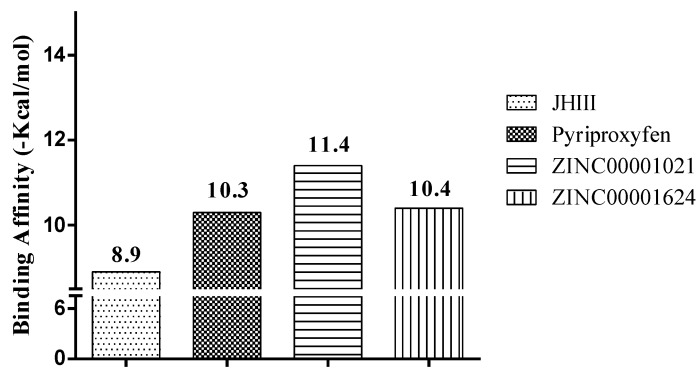
Results of binding affinity of the compounds with the juvenile hormone (PDB ID 5V13).

We observed that of the five compounds submitted to the molecular docking studies, only two show values higher than or equal to the controls used. Regarding the JHIII complex (*Aedes aegypti* organism), the compound ZINC0001021 and ZINC00001624 presented higher value than the controls used (JHIII and pyriproxyfen), with values of −11.4 and −10.4 Kcal/mol, respectively. Results of the affinity values can be observed according to Figure 6. JHIII showed binding affinity of −8.9 kcal/mol, i.e., lower than pyriproxyfen (of −10.3 Kcal/mol). However, the compound ZINC0001021 shows a binding affinity value of −11.4 kcal/mol, i.e., higher than the controls used in molecular docking. Therefore, by comparing the ZINC0001021 compound to the JHIII control, a difference of ±2.5 Kcal/mol was observed, and the others a variation of ±1.0 to ±1.1 Kcal/mol.

With these data, we propose that the compounds are capable of binding to active sites. However, the compound ZINC0001624 has higher affinity to the active site of the human acetylcholinesterase, whereas ZINC0001021 has higher binding affinity to the active site of the mosquito juvenile hormone-binding protein. Compound ZINC00001021 shows similar interactions to the active site of acetylcholinesterase for I40 around the α-helix between amino acid residues Tyr370–Tyr374 and β-sheet with amino acid residues Trp83 and Leu479. The binding affinity value similar to the observed for the control shows that such a compound is a potential insecticide.

**Figure 7:**
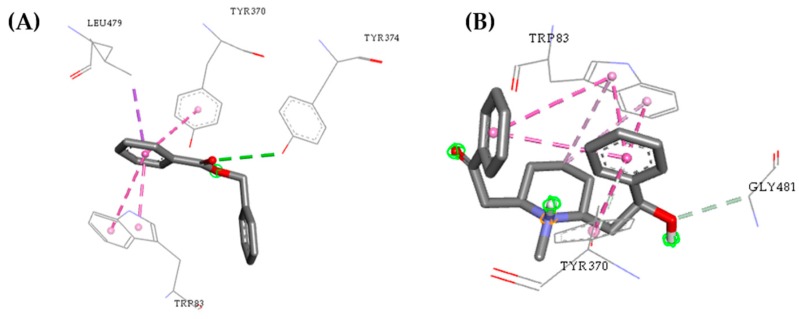
Interactions between the acetylcholinesterase active site and the compounds ZINC00001021 (**A**) and ZINC00001624 (**B**).

The interactions individually observed after docking of compound ZINC0001021 and ZINC0001624 had similar interactions with I40 regarding the acetylcholinesterase active site, located around the α-helix between amino acid residues Tyr370 and β-sheet with amino acid residues Trp83, Leu479, and Gly481, as can be seen in Figure 7.

Compounds with potential insecticidal activity may irreversibly inhibit the production of acetylcholinesterase; this enzyme is responsible for the hydrolysis of acetylcholine (ACh) that terminates the nerve impulse. Inhibition of the acetylcholinesterase enzyme is particularly the initial mechanism for a substance to be considered to have potential insecticide in the larval phase, considering the knowledge cited by several authors [49,50,51,52]. It is essential to observe interactions formed inside the active site of the acetylcholinesterase, in which three important characteristics are present in order to know the mechanism of elucidation of biological action of the enzyme production.

The interactions obtained after molecular docking of the compounds with the amino acid residues Trp71, Trp83, Tyr370, Phe371, and His480 of acetylcholinesterase are similar to those reported in the literature [53,54]. In the compound ZINC0001624, the most significant contributions of the interactions were observed with the docking study performed here, where the contribution of the residues Trp83, Tyr370, and Gly481 to the increase of the binding affinity could thus inactivate the enzyme acetylcholinesterase by competition with the I40 for the AChE active site.

In considering the active site of acetylcholinesterase that binds GNT around the α-helix between amino acid residues Tyr337 and β-sheet with amino acid residues Trp86 and Tyr124, the compound ZINC00001021 had similar interactions. The binding affinity value obtained was similar to the observed for galantamine, pointing out that such a compound is a potential insecticide.

**Figure 8:**
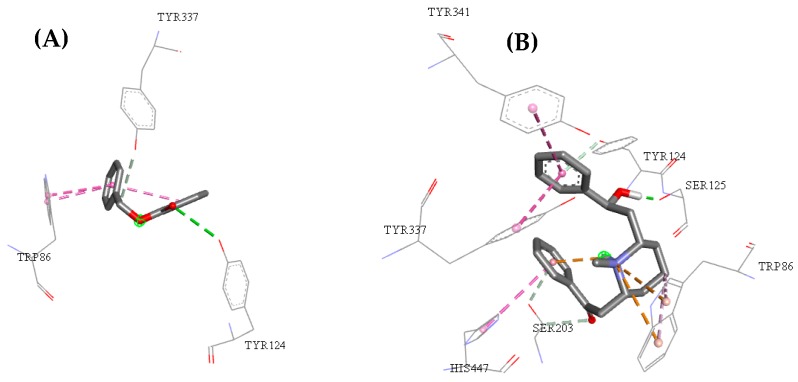
Active site interactions between the human acetylcholinesterase active site and the molecules ZINC00001021 (**A**) and ZINC00001624 (**B**).

Compounds ZINC00001021 and ZINC00001624 had similar interactions to the observed between the acetylcholinesterase active site and GNT, i.e., around the α-helix between amino acid residues Tyr337 and β-sheet with amino acid residues Trp86, Tyr124, Ser125, Ser203, Tyr341, and His447, such as can be seen in Figure 8.

According to Meriç [55], in the AChE active site the catalytic triad (Ser203, Glu334, and His447) is located in the lower portion of the active site, surrounded by three important aspects for the catalytic activity: the acyl pocket (residues Phe295, Phe297, and Phe338), the oxy-anion channel (main residue nitrogen Gly121, Gly122, and Ala204) and the choline binding site (Trp86 and Tyr337). For the compound ZINC00001624, the most significant contributions of the interactions were observed in the docking study, where the contribution of the catalytic triad (represented by Ser203 and His447) and choline binding (Trp86 and Tyr337) for the increase of the binding affinity was observed, thus inactivating the enzyme acetylcholinesterase by competition with the active site with the GNT.

**Figure 9:**
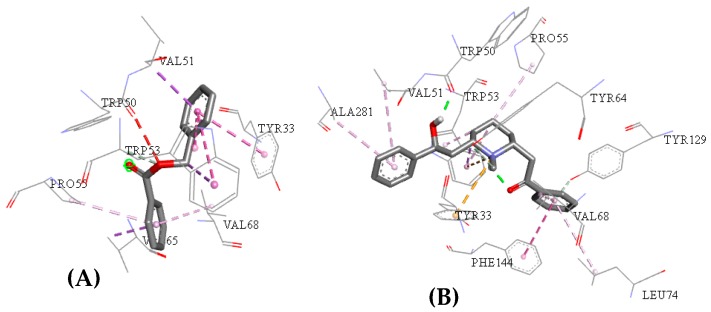
Interactions between the active site of juvenile hormone with the molecules ZINC00001021 (**A**) and ZINC00001624 (**B**).

The individual interactions observed performing docking of molecules ZINC00001021 and ZINC00001624 were similar to the observed for JHIII inside the active site of the juvenile hormone, i.e., around the α-helix between amino acid residues Val68, Typ129, and Phe144 and in β-sheet with Val51, Trp53, and Pro55, as shown in Figure 9.

The growth, development, metamorphosis, and reproduction of insects are under control of juvenile and ecdysteroid hormones, or molting hormones, secreted by specific endocrine glands, corpora allata, and prothoracic glands. The receptors of these two large groups of insect hormones have become targets for neurotoxic pesticides and insecticides. The development of these “biorational” insecticides, such as methoprene and tebufenozide, are based on classical bioassays that measure the agonist activity of these hormones [56].

The crystal structure of the mosquito hormone binding protein (mJHB) bound to JHIII was determined by molecular substitution using an N-terminal polyalanine model of the N-terminal domain of a D7 (salivary odoriferous proteins) [57]. The N-terminal domain of the long D7s binds to vertebrate eicosanoid mediators in *Aedes* and *Anopheles*. In *Aedes* sp., the C-terminal domain binds biogenic amines including serotonin and histamine, while the C-terminal domain of *Anopheles* does not appear to have a small molecule binding site [19]. 

Proteins of this type would bind to small hydrophobic molecules that act on essential physiological processes. In studies, a D7-like protein in the hemolymph of *Ae. aegypti* is a ligand-specific JH binding protein. The crystallographic structure of the JHIII-protein complex reveals a single binding site, and this causes the protein to undergo a conformational change in the hormone load that stabilizes the ligand in the binding pocket [19,57].

A single well-ordered molecule of JHIII (refined occupations 0.91–0.96) was present in the N-terminal domain binding pocket of mJHB. Three molecules of the complex were present in the asymmetric unit of the crystal, and the conformation of the linker was essentially identical in all three [19]. The epoxy of JHIII was located in the center of the domain, while the methyl ester group of the hormone was oriented towards its surface. The epoxy group forms a hydrogen bond with the phenolic hydroxyl of Tyr-129, and the remainder of the isoprenoid chain was surrounded by hydrophobic side chains, including Phe144, Tyr64, Trp53, Val65, Val68, Leu72, Leu74, Val51, and Tyr33.

Molecule ZINC00001021 (**Z21**) showed significant contributions to the binding affinities (−11.4 kcal/mol) calculated using molecular docking, because it conforms to the active site, in which they are represented by amino acids surrounded by hydrophobic side chains including Try33, Val51, Trp53, Val65, and Val68.

Quantitative data on residues, distances, type, and free binding energies (ΔG) between the promising compounds and the insect/human acetylcholinesterase and juvenile hormone enzyme can be seen in Tables S1–S3 (see Supplementary Materials). It is possible to verify that the reference molecules (I40, GNT, and JHIII ligand) and the other ligands had an increase in the number of interactions that resulted in the decrease of the free binding energy, indicating a higher degree of spontaneity of the interactions. We have observed that interactions with the residues Trp83, Tyr370, Tyr374, and Leu479 are common and most recurrent for all the compounds used here for molecular docking. Interaction with the Trp83 and Tyr370 residue is also common in most molecules, however less recurrent than Phe371, Gly481, and His480, as shown in Table S1, Supplementary Materials. These results make it possible to infer that these two residues play a key role in the potential insecticidal activity.

Regarding to the molecule ZINC0001624 (**Z24**), with higher free energy (−12.00 kcal/mol), hydrogen bonding interactions occur with His480, as well as pi-alkyl with Trp83, and they are similar to those that occur with I40, indicating that such a compound has a potential insecticidal action, as well as it could interact more effectively with the enzyme active site. The most promising molecule ZINC0001624 shows good results by the docking analysis, because it has a high value of free energy, which contributes to its greater stability when interacting with the active site of the insect acetylcholinesterase.

Interactions with residues Trp86, Tyr124, Tyr337, and His447 are common and most recurrent in all compounds investigated here. The interaction with the Ser125 residue is also common in most molecules, however, less recurrent than Tyr133 and Ser203.

Molecule ZINC0001624 (**Z24**) shows free energy of −10.40 kcal/mol, hydrogen bonding interactions with Ser203, as well as pi-alkyl interactions with Trp86 in a similar way to those observed for GNT, indicating that such a molecule has a potential insecticidal action, for inhibiting the production of the enzyme acetylcholinesterase, as shown in Table S2, Supplementary Materials. The most promising molecule is ZINC0001624 because it shows a free energy value (ΔG) higher than the observed for GNT, molecules complexed with acetylcholinesterase, and used here in the molecular docking.

Binding affinity and ΔG values for the insect and human acetylcholinesterase enzymes were matched, since there was homology and high sequence similarity shared among *Ae. aegypti*, *D. melanogaster*, and *H. sapiens*. Inhibitor selectivity was due to Tyr71, Tyr73, Glu80, and Asp375, which in vertebrates are Asp, Gln, Ser, and Gly, according to studies of Harel et al. [48]. 

Significant differences in binding affinity values and ΔG were due to a 50% reduction in the active site cleft of the insect acetylcholinesterase, compared to the human enzyme, according to the literature [9,48], thus corroborating the values obtained with molecular docking.

Molecule ZINC00001624 (**Z24**) also had significant affinities calculated for residues Phe144, Tyr64, Trp53, Val65, Val68, Leu72, Leu74, Val51, and Ty33. However, comparing the distances of the key interactions that occur inside the pocket (including Try33, Val51, Trp53, Val65, and Val68) the interaction forces were more intense for ZINC00001021 (**Z21**), with higher affinity values and ΔG value of −8.13 kcal/mol, as shown in Table S3, Supplementary Materials.

The most common interactions observed for compounds docked here with juvenile hormone were with the amino acid residues Val51, Trp53, Pro55, and Val68. Interactions of the residues Tyr129 and Phe144 occurred for compound ZINC00001624 and control JH, and the less common interactions occurred with Leu74.

### 2.4. Toxicity Risk Assessment

Semi-volatile organic compounds (SVOCs), such as pesticides present in the atmosphere, are known to be simultaneously present in both gas and particulate phases. The physical distribution is related to the physicochemical properties of the compound in question, such as vapor pressure and water solubility. This is also influenced by environmental conditions, especially temperature, humidity, and particulate matter concentration [58].

Pyriproxyfen is used today as vector control in agriculture, since the targets have little resistance to their effect [59]. Lethal dose 50 (LD_50_) values are reported in (mg/kg) according to the World Health Organization (WHO) pesticide classification ranges, which identify five groups based on LD_50_ oral data (Hazard Categories) in the rat [60,61,62,63]. The severity of the effects depends on the classification of the pesticides and the dose of exposure. The molecules used in this step do not violate the Lipinski rule, as can be seen in Table 1. The values of oral toxicity predictions of the molecules can be seen in Table 4.

The oral toxicity values defined for the molecules were 2000 (mg/kg) for pyriproxyfen (control) and 1000 (mg/kg) ZINC00001021, classifying them as belonging to class IV (300 < LD_50_ ≤ 2000), harmful if swallowed. The molecule ZINC00001624 with an LD_50_ value of 349 (mg/kg), being classified in Class III (50 < LD_50_ ≤ 300), considered to be toxic if swallowed. The toxicity study corroborates the LD_50_ values determined for the molecules, including the control for not being alert to humans, as shown in Table 2.

### 2.5. Structure–Activity Relationship of the Promising Molecule

**Figure 10:**
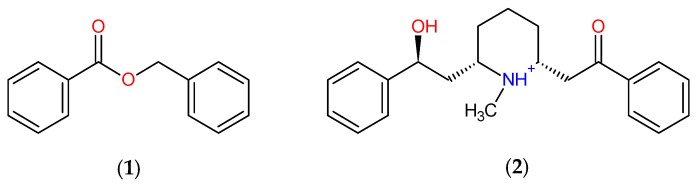
Promising molecules ZINC00001021 (**1**) and ZINC00001624 (**2**) obtained after virtual screening.

Molecule (**1**) in its structural structure has a benzoate grouping which contributes to the insecticidal potential. The class causes reversible inhibition of the enzyme acetylcholinesterase, present in the parasympathetic and sympathetic ganglia, in the parasympathetic muscarinic terminal junctions, sympathetic fibers located in the sweat glands, and in the nicotinic receptors in the skeletal neuromuscular junction [64,65,66,67,68], as shown in Figure 10. Molecule (1) is the major component of the essential oil of the species *Cananga odorata* with moderate insecticidal activity with LD_50_ at 52.96 ppm against the immature stage of *Ae. aegypti*, according to studies of Tan et al. (2015) [69]. 

Benzyl benzoate is a product of natural origin and is commonly isolated from aromatic plants. Aromatic compounds are classified into three groups: monoterpenes, sesquiterpenes, and aliphatic compounds (alkanes, alkenes, aldehydes, ketones, acids, and alcohols) and they have potential efficiency in the control of insects compared to synthetic pesticides, since the high volatility essential oils makes them good fumigant agents against insect pests [70]. Mostafiz et al. (2018) [71] studied insecticidal and repellent effects (biopesticide) of methyl benzoate (BM) against *Bemisia tabaci* (Gennadius) (Hemiptera: Aleyrodidae) and showed that the results suggest that the BM has a strong potential as a biopesticide that is environmentally friendly for the control of *B. tabaci* and is easily biodegradable.

Literature data from Yamamoto (1999) [72] show that more than 10,000 alkaloids have been described and are one of the most diverse and prominent groups of natural products with pharmacological and toxicological importance. Crude extracts obtained from plants containing bioactive alkaloids; insecticides have an important role in reducing insects in agriculture and public health.

Lobeline is an alkaloid extracted from the leaves of *Lobelia inflata* (Campanulaceae), native to the eastern and southeastern region of North America. The presence of alkaloids may be signaled to a variety of biological activities, for example, ametine (amebicide), atropine, hyoscyamine and scopolamine (anticholinergic), reserpine and protoveratine A (antihypertensive), quinine (antimalarial), and galantamine (Alzheimer’s treatment). There is little information available about how lobeline affects insects, although it has been reported that it holds the feeding of caterpillars and bees. The lobeline pharmacology has proven to be complex since it shares some of the agonist properties of nicotine and, like nicotine, binds nicotinic acetylcholine receptors (*n*AChRs) with high affinity [73]. 

Molecule (**2**) exhibits the presence of the alcohol, amine, and ketone groups. The amine group is classified into the class of secondary metabolite belonging to the alkaloid family which has a proven action inhibiting insect acetylcholinesterase [68,69], as shown in Figure 10.

## 3. Materials and Methods 

### 3.1. Template Compound

Since the bioactive conformation of pyriproxyfen has not yet been structurally determined, its more rigid conformation based on the available crystallographic structure according to Kang et al., (2015) [74], was used as the input file for the analysis of virtual screening by structural and electrostatic similarity, with the rapid overlay of chemical structures (ROCS) and electrostatic similarity (EON) software [75,76,77].

### 3.2. Generation of Conformers Library in Database

In this present work, a commercial database was used to perform the virtual screening step. The ZINC database (http://zinc.docking.org/) contains more than 35 million compounds, and it is regularly updated and can be used free of charge on the web-server or by download. For each molecule of the database, 300 conformers were generated using the MMFF94 force field implemented in OMEGA software (OpenEye Scientific Software, Santa Fé, NM. http://www.eyesopen.com), on a computer with Intel Core i7 2.4 GHz processor using Windows 7 Professional operating system. A strain tension (energy difference of a respective conformer and the global minimum energy of the molecule) of up to 9 kcal/mol and a root mean square deviation (RMSD) of 0.6 Å were also used [76,77].

### 3.3. Virtual Screening

#### 3.3.1. Rapid Overlay of Chemical Structures (ROCS)

The ZINC database was used to select molecules via virtual screening, in which the shape was approximated by atom-centered overlapping Gaussians and was used to calculate the maximum intersection of the volume of two molecules [77]. In this study, the algorithm (Gaussian functions) implemented in the ROCS software (https://www.eyesopen.com/rocs) was used to generate and punctuate the three-dimensional database overlays with the reference structure of pyriproxyfen and thus to obtain the top-ranked 2000 structures [76,77].

#### 3.3.2. Electrostatic Similarity (EON)

With the use of the EON software (https://www.eyesopen.com/eon), the electrostatic Tanimoto indexes of the chemical structures of the selected molecules in the database and the structure of the pyriproxifen were calculated, in addition to calculations of partial loads to minimize energy (MMFF94) [76,77]. The electrostatic classification was based on an electrostatic Tanimoto score, which varies from one (identical) to negative values resulting from the overlapping of positive and negative charges. In this study, a single lowest-energy conformer of pyriproxyfen for all the electrostatic comparisons was used (more rigid conformation, based on the available crystallographic structure). The output files were grouped according to the scores, and the results were classified based on the “ET_combo” analogous to “ComboTanimoto”. At the end, only the “Top-ranked 100 molecules/base” were selected.

### 3.4. In silico Pharmacokinetic and Toxicological Properties

The molecules selected from the previous step were submitted to the QikProp software in order to obtain pharmacokinetic properties [78]. The parameters generated by the software allowed us to select the candidate having a parameter of 95% of approximation with pharmacokinetic characteristics of drugs already described in the literature, giving reliability to the generated data [79]. The toxicity profile of the molecules was performed using DEREK software. DEREK is in silico toxicological prediction software used for drug design purposes [80,81,82,83]. 

### 3.5. Biological Activity Predictions of the Compounds from Virtual Screening

Predictions of biological activity were performed using the PASS online web server, available at http://www.pharmaexpert.ru/passonline [84]. Using PASS, it was possible to discover biological effects of a compound based entirely in the formula using MNA descriptors (multilevel neighboring of atoms), suggesting that biological activity is in the function of their chemical structure [85,86,87]. Only molecules with insecticidal and anticholinesterasic activities indications were selected at this stage.

### 3.6. Molecular Docking Simulations

At this step, only the top-ranked molecules with satisfactory results regarding the pharmacokinetic, toxicological, and biological activity predictions were selected for the molecular docking simulations, in order to evaluate the energy function scores through the free energy value (ΔG) of the interaction of ligands derived from the ligand-based virtual screening, as well as analysis of the conformations and binding mode and binding affinity with the targets used here.

#### 3.6.1. Selection of Enzymes and Inhibitors Structures

As insecticides can act in different sites, the two pathways of action mechanism are the highlights: acetylcholinesterase (AChE) and juvenile hormone (JH) enzymes. AChE is a significant target, since carbamates and organophosphates are classes of pesticides capable of inhibiting AChE. Thus, despite the high toxicity of these substances, they are still widely used in agriculture and domestic use [88] and show high resolution crystallographic structure AChE, isolated and in complexes with ligands, thus justifying the use of this enzyme at this step. Similarly, JHIII is a key regulator of insect development and breeding. In adult mosquitoes, it is essential for ovary maturation and normal male reproductive behavior, but how the distribution and activity of JH are regulated is unclear after secretion [20].

An additional comparative study of molecular modeling was performed with the crystalline structure of human acetylcholinesterase complexed with (−)-galantamine (GNT), in order to evaluate free energy, interactions with amino acid residues, and binding affinity. The insect and human AChEs share high sequence similarity, according to data found in the literature [9,10,11,12,13,14]. Thus, the lack of crystallographic structure of complex acetylcholinesterase elucidated for *Aedes aegypti* was motivated by the choice of the targets investigated here.

**Figure 11:**
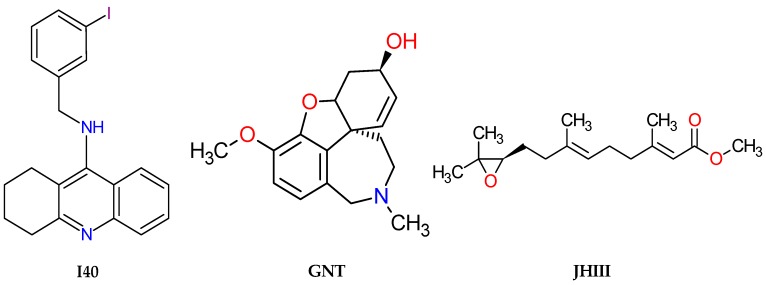
Structures of the AChE inhibitors, 9-(3-iodobenzylamino)-1,2,3,4-tetrahydroacridine (I40), (−)-galanthamine (GNT) and methyl(2E,6E)-9-[(2R)-3,3-dimethyloxiran-2-yl]-3,7-dimethylnona-2,6-dienoate (JHIII), used in the present work.

The crystallographic structure of the *Drosophila melanogaster* acetylcholinesterase (AChE) complexed with tacrine derivative 9-(3-iodobenzylamino)-1,2,3,4-tetrahydroacridine (I40) was downloaded from the Protein Data Bank (PDB), with PDB ID 1QON and 2.7 Å resolution [48]. The crystallographic structure of the recombinant human acetylcholinesterase (*h*AChE) complexed with (−)-galantamine (GNT), eluted by X-ray diffraction was downloaded from the Protein Data Bank (PDB), with PDB ID 4EY6 and 2.4 Å resolution [84,85]. The crystallographic structure of juvenile hormone complexed with methyl (2E,6E)-9-[(2R)-3,3-dimethyloxiran-2-yl]-3,7-dimethylnona-2,6-dienoate, (JHIII), was downloaded with the PDB ID 5V13 and 1.87 Å resolution [20]. The I40, GNT, and JHIII and pyriproxyfen compounds were used here as control inhibitors in the molecular docking studies, based on a well-established protocol developed by our research group [89,90,91,92,93]. The structures of the ligands complexed with acetylcholinesterase and juvenile hormone, can be respectively seen in Figure 11.

#### 3.6.2. Docking Study with AutoDock 4.2/Vina 1.1.2 via Graphical Interface PyRx (Version 0.8.30)

Inhibitors and protein structure used in molecular docking studies performed here were prepared using the Discovery Studio 5.0 software [94]. AChE (*D. melanogaster* and *Homo sapiens*) and juvenile hormone (*Aedes aegypti*) in complex with specific ligands were used in AutoDock 4.2/Vina 1.1.2 and graphical interface PyRx version 0.8.30 (https://pyrx.sourceforge.io), respectively. The validation of molecular docking of the ligand was performed by comparison between the crystallographic ligand and the best one conformation obtained with molecular docking (structure of PDB ID: 1QON, 4EY6, and 5V13), based on the RMSD value.

Coordinates x, y, and z of the receptors were determined according to the middle region of the active site. The coordinates used here for the center of the grid can be seen in Table 5. An energy function score was used to evaluate the binding free energy (ΔG) of the interaction of the ligands with the amino acid residues of the receptors. The conformational analysis was also taken into account for the selection of the best binding free energy for binding affinity calculations via AutoDock 4.2/Vina 1.1.2. Visualizations as well as distance measures of interactions between inhibitors and enzymes performed using Discovery Studio 5.0.

### 3.7. Toxicity Risk Assessment

ProTox, a virtual lab for prediction of toxicities of small molecules as well as a useful tool to identify any undesirable toxic properties of our molecules was used [61] (see http://tox.charite.de/protox_II/). The prediction was based on functional group similarity for the query molecules with the in vitro and in vivo contained in the database. Toxic properties such as LD_50_ values in mg/kg and toxicity class were determined.

## 4. Conclusions

Recent research has shown great interest in the mechanism of chemical action of novel biocidal agents. To date, an insecticide has not yet been developed that acts directly on the inhibition of the enzyme acetylcholinesterase and juvenile hormone, and the approach to rational design may mean a new option to obtain new compounds with potential biocidal activity. Initial molecular docking analysis allowed us to select only molecules that competed with the active site of the enzyme acetylcholinesterase and juvenile hormone. Results obtained here were considered satisfactory, because they allow the selection of structures with good pharmacokinetic and toxicological properties, which are important for selection of more efficient molecules against the disease in question.

Molecules ZINC00001021 and ZINC00001624 showed the best results after the molecular docking analysis with the acetylcholinesterase and juvenile hormone enzymes. The inhibitor-enzyme interactions observed for these molecules were similar to those observed for the reference compounds (GNT, JHIII, and pyriproxyfen) with the same biological target. In addition, lower ΔG values were found for the two molecules, after virtual screening and ΔG, in comparison to the controls.

Molecules ZINC00001021 and ZINC00001624, in general, showed excellent predictions in all the steps of the study, and such compounds may be indicated as the most promising insecticidal agents, which are resulting from virtual screening. Our research group intends to build an AChE model for *Ae. aegypti*, through homology modeling studies, as well as performing biological assays with the molecules obtained in this paper, in order to confirm such predictions in silico.

## Figures and Tables

**Table 1:** Pharmacokinetic properties of selected molecules.

Molecules	^a^ Star	^b^ CNS	^c^ MW	^d^ QP logKp	^e^ HBD	^f^ HBA	^g^ R5	^h^ R3
Normal range	0–5	−2 to +2	<500	−8 to −1	<5	<10	Max. 4	Max. 3
Pyriproxyfen	1	1	321.4	0.8	0	4	1	0
ZINC11616655	0	0	376.5	−1.6	1	3	0	1
ZINC13537284	0	0	294.4	−1.7	2	5	0	0
ZINC00073711	0	1	201.2	−1.8	1	3	0	0
ZINC00001021	0	0	212.2	−0.6	0	2	0	0
ZINC11616399	0	0	384.5	−2.3	0	4	0	1
ZINC01530753	0	1	356.1	−2.3	0	4	0	0
ZINC01530718	0	1	267.4	−3.0	3	4	0	0
ZINC11616398	0	0	384.5	−2.2	0	4	0	1
ZINC00000257	0	0	295.4	−4.1	1	4	0	0
ZINC04363405	0	0	414.6	−2.9	0	4	0	1
ZINC03831238	0	0	299.4	−3.3	4	3	0	0
ZINC12504271	0	0	366.8	−2.4	0	3	0	1
ZINC00538483	0	1	371.9	−3.4	1	6	0	0
ZINC00001624	0	1	337.5	−2.9	2	3	0	0

^[a]^ Number of computed properties which fall outside the required range for 95% of known drug; ^[b]^ activity in the central nervous system^; [c]^ molar weight; ^[d]^ the predicted skin permeability; ^[e]^ number of hydrogen bonds donated by the molecule; ^[f]^ number of hydrogen bonds accepted by the molecule; ^[g]^ number of violations of Lipinski’s ‘rule of five’; ^[h]^ number of violations of Jorgensen’s ‘rule of three’.

**Table 2:** Predictions of the toxicological properties of molecules.

Molecules	Prediction	Alert
Pyriproxyfen	-	No alert
ZINC11616655	Hepatotoxicity	Plausible
	Skin sensitization	
	Teratogenicity	
	Estrogenicity	
ZINC13537284	-	No alert
ZINC00073711	Hepatotoxicity	Plausible
	Teratogenicity	
ZINC00001021	-	No alert
ZINC11616399	Hepatotoxicity	Plausible
	Skin sensitization	
	Teratogenicity	
	Estrogenicity	
ZINC01530753	Carcinogenicity	Plausible
	Chromosome damage	
	Skin sensitization	
ZINC01530718	-	No alert
ZINC11616398	Hepatotoxicity	Plausible
	Skin sensitization	
	Teratogenicity	
	Estrogenicity	
ZINC00000257	-	No alert
ZINC04363405	Skin sensitization	Plausible
ZINC03831238	hERG channel inhibition	Plausible
	Skin sensitization	
ZINC12504271	Skin sensitization	Plausible
ZINC00538483	hERG channel inhibition	Plausible
	Skin sensitization	
ZINC00001624	-	No alert

**Table 3:** Biological activity prediction of the compounds selected by virtual screening.

Molecules	Pa ^a^	Pi ^b^	Biological Activity
Pyriproxyfen	0.586	0.003	Insecticide
I40	0.025	0.005	Acetylcholine transporter inhibitor
GNT	0.376	0.154	Acetylcholine neuromuscular blocking agent
JHIII	0.336	0.011	Insecticide
ZINC13537284	-	-	-
ZINC00001021	0.444	0.005	Insecticide
ZINC01530718	-	-	-
ZINC00000257	-	-	-
ZINC00001624	0.450	0.005	Acetylcholine antagonist
	0.433	0.044	Acetyl esterase inhibitor

^a^ Pa = probability to be active; ^b^ Pi = probability to be inactive.

**Table 4:** Oral toxicity prediction results for input compound.

Molecules	Predicted LD_50_ (mg/kg)	Predicted Toxicity Class ^[a]^
Pyriproxyfen (Control)	2000	IV
I40	200	III
GNT	19	II
JHIII	5000	IV
ZINC00001021	1000	IV
ZINC00001624	349	III

^[a]^ Class I: fatal if swallowed (LD_50_ ≤ 5); Class II: fatal if swallowed (5 < LD_50_ ≤ 50); Class III: toxic if swallowed (50 < LD_50_ ≤ 300); Class IV: harmful if swallowed (300 < LD_50_ ≤ 2000); Class V: may be harmful if swallowed (2000 < LD_50_ ≤ 5000); Class VI: non-toxic (LD_50_ > 5000).

**Table 5:** Data from protocols used here for molecular docking validation.

Enzyme	Inhibitor	Coordinates of the Grid Center	Grid Size (Points)
AChE (PDB code: 1QON)	9-(3-Iodobenzylamino)-1,2,3,4-Tetrahydroacridine	X = 33.4862 Y = 67.9151 Z = 9.4399	35 x 34 y 31 z
AChE (PDB code: 4EY6)	(−)-galanthamine	X = 9.090 Y = −60.485 Z = −23.703	32 x 38 y 36 z
Juvenile hormone (PDB code: 5V13)	methyl (2E,6E)-9-[(2R)-3,3-dimethyloxiran-2-yl]-3,7-dimethylnona-2,6-dienoate	X = −213.788 Y = 1.653 Z = 352.848	40 x 44 y 36 z

## Supplementary Materials

The following are available online at http://www.mdpi.com/1424-8247/12/1/20/s1, Table S1. Type of interactions observed between acetylcholinesterase and the most promising molecules, Table S2. Type of interactions observed between human acetylcholinesterase and the most promising molecules, Table S3. Binding receptor interaction data juvenile hormone of the most promising molecules.
