# Supplementary material for: Identification of Potential Inhibitors from Pyriproxyfen with Insecticidal Activity by Virtual Screening
Source: Pharmaceuticals (Basel). 2019 Jan 25;12(1):20. doi: 10.3390/ph12010020 (PMC6469432; doi:10.3390/ph12010020)
Supplement: Supplementary file 1 [file pharmaceuticals-12-00020-s001.pdf]

pharmaceuticals

Article

Identification of Potential Inhibitors from Pyriproxyfen with Insecticidal Activity by virtual screening

Ryan da Silva Ramos [1,2,3], Josivan da Silva Costa [2,3], Rai Campos Silva [2,4], Glauber Vilhena da Costa [2], Alex Bruno Lobato Rodrigues [1,2], Érica de Menezes Rabelo [1], Raimundo Nonato Picanço Souto [5], Carlton Anthony Taft [6], Carlos Henrique Tomich de Paula da Silva [3,4], Joaquín Maria Campos Rosa [7], Cleydson Breno Rodrigues dos Santos [1,2,4*], Williams Jorge da Cruz Macêdo [1,2,3]

[1] Postgraduate Program in Biotechnology and Biodiversity-Network BIONORTE, Federal University of Amapá, Macapá, 68903-419 Amapá, Brazil, ryanquimico@hotmail.com
[2] Laboratory of Modeling and Computational Chemistry, Department of Biological and Health Sciences, Federal University of Amapá, 68902-280, Macapá, AP, Brazil; josivan.chemistry@gmail.com, camposchemistry@gmail.com, vilhenac@hotmail.com, alexrodrigues.quim@gmail.com, ericamrabelo@gmail.com
[3] Laboratory of Molecular Modeling and Simulation System, Federal Rural University of Amazônia, Capanema, Pará, 68700-030, Brazil; williamsmacedo@yahoo.com.br
[4] Computational Laboratory of Pharmaceutical Chemistry, Faculty of Pharmaceutical Sciences of Ribeirão Preto, 14040- 903, São Paulo, Brazil; tomich@fcfrp.usp.br
[5] Laboratory of Arthropoda, Federal University of Amapá, 68902-280, Macapá, AP, Brazil; nonatoiepa@hotmail.com
[6] Brazilian Center for Physical Research, Urca, 22290-180, Rio de Janeiro, Brazil; catff@terra.com.br
[7] Department of Pharmaceutical Organic Chemistry, University of Granada, Granada, 18071, Spain; jmcampos@ugr.es
* Correspondence: breno@unifap.br; Tel.: +55-096-4009-2699

SUPPLEMENTARY MATERIAL

Table S1. Type of interactions observed between acetylcholinesterase and the most promising molecules.

Table S2. Type of interactions observed between human acetylcholinesterase and the most promising molecules.

Table S3. Binding receptor interaction data juvenile hormone of the most promising molecules.

Table S1. Type of interactions observed between acetylcholinesterase and the most promising molecules.

Molecular target	Residues	Distance (Å)	Type	ΔG (kcal/mol)
	Trp71	4.10	Pi-Pi Stacked	
	Trp83	3.91	Pi-Pi Stacked	
	Trp83	3.55	Pi-Pi Stacked	
	Trp83	3.92	Pi-Pi Stacked	
	Trp83	4.28	Pi-Alkyl	
I40 vs 1QON	Tyr370	3.86	Pi-Pi Stacked	-12.76
	Tyr370	4.15	Pi-Pi Stacked	
	Phe371	5.23	Pi-Alkyl	
	Tyr374	4.95	Pi-Pi T-shaped	
	Leu379	5.44	Pi-Alkyl	
	His480	2.08	Convent. Hydrogen Bond	
	Trp83	3.63	Pi-Pi Stacked	
	Trp83	4.47	Pi-Pi Stacked	
Z21 x 1QON	Tyr370	4.33	Pi-Pi Stacked	-7.70
	Tyr374	3.30	Convent. Hydrogen Bond	
	Leu479	3.90	Pi-Sigma	
	Trp83	3.79	Pi-Pi Stacked	
	Trp83	3.54	Pi-Pi Stacked	
Z24 vs 1QON	Trp83	4.67	Pi-Alkyl	-12.00
	Tyr370	3.36	Pi-Donor Hydrogen Bond	
	Tyr370	4.22	Pi-Pi Stacked	
	Gly481	3.19	Carbon Hydrogen Bond	

Table S2. Type of interactions observed between human acetylcholinesterase and the most promising molecules

Molecular target	Residues	Distance (Å)	Type	ΔG (kcal/mol)
GNT vs 4EY6	Trp86	4.40	Pi-Alkyl	-9.79
	Trp86	4.72	Pi-Alkyl	
	Trp86	5.25	Pi-Alkyl	
	Gly121	3.96	Amide-Pi Stacked	
	Tyr124	3.58	Carbon Hydrogen Bond	
	Glu202	1.76	Conventional Hydrogen Bond	
	Ser203	2.80	Conventional Hydrogen Bond	
	Tyr337	5.42	Pi-Alkyl	
Z21 x 4EY6	Trp86	4.45	Pi-Pi Stacked	-6.51
	Trp86	3.97	Pi-Pi Stacked	
	Tyr124	3.28	Conventional Hydrogen Bond	
	Tyr337	3.75	Carbon Hydrogen Bond	
Z24 vs 4EY6	Trp86	4.35	Pi-Cation	-10.40
	Trp86	4.48	Pi-Cation	
	Trp86	4.86	Pi-Alkyl	
	Tyr124	3.14	Pi-Donor Hydrogen Bond	
	Ser125	1.83	Conventional Hydrogen Bond	
	Ser203	3.10	Carbon Hydrogen Bond	
	Ser203	3.30	Pi-Donor Hydrogen Bond	
	Tyr337	5.18	Pi-Pi T-shaped	
	Tyr341	5.15	Pi-Pi T-shaped	
	His447	4.80	Pi-Pi T-shaped	

Table S3. Binding receptor interaction data juvenile hormone of the most promising molecules.

**Molecular docking**	**Residues**	**Distance (Å)**	**Type**	**ΔG (kcal/mol)**
	Leu37	4.66	Alkyl	
	Val51	4.59	Alkyl	
	Val51	4.83	Alkyl	
	Trp53	3.72	Pi-sigma	
	Trp53	4.80	Pi-Alkyl	
	Trp53	4.56	Pi-Alkyl	
JHIII vs 5V13	Trp53	4.54	Pi-Alkyl	-8.35
	Pro55	4.13	Alkyl	
	Val68	4.89	Alkyl	
	Tyr129	4.91	Pi-Alkyl	
	Tyr133	5.02	Pi-Alkyl	
	Phe144	4.39	Pi-Alkyl	
	Ala281	4.36	Alkyl	
	Tyr33	4.52	Pi-Pi Stacked	
	Trp50	2.91	Unfavorable Acceptor-Acceptor	
	Val51	3.63	Pi-Sigma	
Z21 vs 5V13	Trp53	3.62	Carbon Hydrogen Bond	-8.13
	Trp53	3.52	Pi-Sigma	
	Trp53	5.00	Pi-Pi T-shaped	
	Pro55	4.92	Pi-Alkyl	
	Val65	3.87	Pi-Sigma	
	Tyr33	4.32	Pi-Cation	
	Trp50	1.97	Conventional Hydrogen Bond	
	Trp53	4.50	Pi-Cation	
	Trp53	4.15	Pi-Alkyl	
Z24 vs 5V13	Trp53	3.41	Pi-sigma	-11.17
	Trp53	4.35	Pi-Alkyl	
	Pro55	4.49	Alkyl	
	Tyr64	3.21	Carbon Hydrogen Bond	
	Tyr129	3.28	Pi-Donor Hydrogen Bond	
